# Earlier detection of SARS‐CoV‐2 infection by blood RNA signature microfluidics assay

**DOI:** 10.1002/ctd2.47

**Published:** 2022-07-10

**Authors:** Antonio Cappuccio, Jennifer Geis, Yongchao Ge, Venugopalan D. Nair, Naveen Ramalingam, Weiguang Mao, Maria Chikina, Andrew G. Letizia, Stuart C. Sealfon

**Affiliations:** ^1^ Department of Neurology Icahn School of Medicine at Mount Sinai New York New York USA; ^2^ Fluidigm Corporation San Francisco California USA; ^3^ Department of Computational and Systems Biology School of Medicine University of Pittsburgh Pittsburgh Pennsylvania USA; ^4^ Naval Medical Research Center Silver Spring Maryland USA

**Keywords:** COVID‐19, infection diagnosis, host response assay

Dear Editor,

Early SARS‐CoV‐2 diagnosis is a key nonpharmacological strategy to contain the current pandemic. Nucleic acid amplification tests (NAATs), the reference standard for SARS‐CoV‐2 diagnosis, are poorly sensitive during the first 4 days after infection, with false negative rates estimated in the range 67–100%.^1^ Here, we implemented a new assay that shows increased sensitivity to SARS‐CoV‐2 infection during the early window of NAAT false negativity.

Host response assays (HRAs) are emerging as a new paradigm for infection diagnosis,^2^ recently implemented to discriminate viral from bacterial infections,^3^, ^4^ and to detect early respiratory viral illnesses.^5^ Unlike NAATs that target viral genetic material, HRAs target transcriptional alterations in the host blood. These alterations may become detectable by RT‐PCR as early as 12 hours after viral challenge.^6^ Given the potentially higher sensitivity early in infection, we set out to implement the first HRA for SARS‐CoV‐2 diagnosis.

Our work leveraged the COVID‐19 Health Action Response for Marines (CHARM), a prospective study that identified incident SARS‐CoV‐2 infection among US Marine recruits from May 12 to November 5 2020.^7^, ^8^ The cohort included 3249 predominantly young, male participants. Participants were typically tested by an FDA‐approved NAAT for SARS‐CoV‐2 three times during an initial 2‐week quarantine, and then biweekly for 6 weeks during basic training (Figure 1A). Most infected participants were asymptomatic at the first positive NAAT and none required hospitalization. During basic training, 45.1% of participants showed a SARS‐CoV‐2 NAAT positive result at one or more time points. The high infection rate, along with the longitudinal design, made the CHARM study highly instrumental for benchmarking a new SARS‐CoV‐2 diagnostic assay.

**FIGURE 1:**
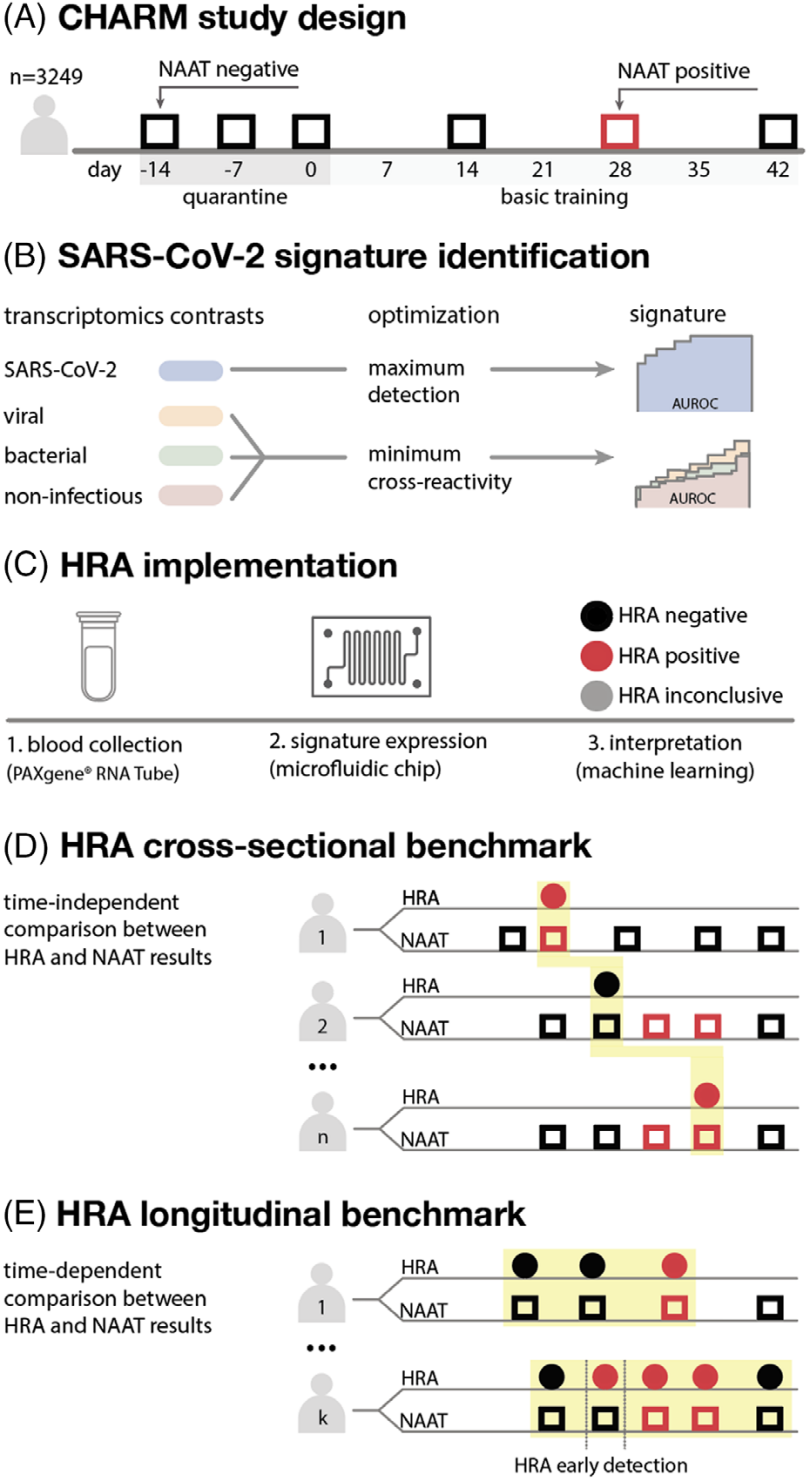
Implementing a SARS‐CoV‐2 host response assay (A) To develop and benchmark a SARS‐CoV‐2 host response assay, we leveraged the CHARM study, a longitudinal study involving a SARS‐CoV‐2 outbreak in a platoon of marine recruits. During the study, participants were serially tested for SARS‐CoV‐2 . (B) Our first step was to identify a host transcriptional response of 41 genes specific for SARS‐CoV‐2 infection, by analyzing a compendium of public COVID‐19 and non‐COVID‐19 studies. The goal of the analysis was to maximize COVID‐19 detection, while minimizing cross‐reactivity with other viral and bacterial infections, and with potential confounders. (C) Next, we implemented a host response assay that takes as input a blood sample, measures the expression levels of the identified signature genes on a microfluidic chip, and returns a sample interpretation based on a machine learning classifier. (D) Schematic representation of a cross‐sectional benchmark aimed at comparing HRA and NAAT results for different participants at random time points. (E) Schematic representation of a longitudinal benchmark aimed at comparing HRA and NAAT repeated measures over time for the same participants. The longitudinal benchmark indicated an earlier detection of SARS‐CoV‐2 by the host response assay compared to an FDA‐approved NAAT

The strategy to develop a SARS‐CoV‐2 HRA followed four main steps (Figure 1B‐E): (1) bioinformatics‐driven identification of a SARS‐CoV‐2 host response signature; (2) technical implementation; (3) cross‐sectional benchmark, by comparing HRA and NAAT results from different participants at randomly selected time points; (4) longitudinal benchmark, by comparing HRA and NAAT repeated measures over time for the same participants.

The first challenge we faced was to identify a host transcriptional response specific for SARS‐CoV‐2 infection. We aimed to find a compact set of 40–50 genes whose expression in the blood would indicate SARS‐CoV‐2‐infection, but not related infections such as influenza. To address this problem, we curated a compendium of public blood transcriptomes from 15 COVID‐19 studies and from 112 studies on a wide variety of viral and bacterial infections (Figure 1B, Supporting information). Furthermore, the compendium included transcriptomes on COVID‐19 comorbidities (e.g., obesity, hypertension) and risk factors (e.g., age, sex) that might act as potential confounders. Applying a combination of meta‐analysis and optimization techniques to the data compendium, we identified 41 genes that together provided robust SARS‐CoV‐2 detection (receiver operator curve (ROC) area under the curve (AUC) 0.7–0.9), and low cross‐reactivity with other infections and confounding factors (ROC AUC ≤0.5).

Next, we implemented a HRA with three main components: whole blood collection through a PAXgene Blood RNA Tube (BD Biosciences, San Jose, CA, USA); measurement of the expression levels of the 41 transcripts on an integrated fluidic circuit; sample interpretation through a machine learning algorithm (Figure 1C, Supporting information). The algorithm was based on a regularized logistic regression classifier, taking as input the combined expression levels of the 41 transcripts measured in a blood sample, and returning as output the sample interpretation in one of the following classes: SARS‐CoV‐2 positive; SARS‐CoV‐2 negative; inclusive, in case of highly uncertain interpretation. The algorithm was developed using a training set of 245 SARS‐CoV‐2 positive and 296 SARS‐CoV‐2 negative samples from the CHARM study. To control for viral cross‐reactivity, the training set included 63 blood samples from subjects in a vaccine trial after H3N2 influenza virus challenge.^9^ During algorithm training, the influenza samples were treated as SARS‐CoV‐2 negative. We performed extensive tests to ensure that the machine learning‐generated interpretation calls were highly reproducible across sample technical replicates.

We first assessed the HRA performance in a cross‐sectional way (Figure 1D, Supporting information). We extracted samples from the SARS‐CoV‐2 positive (n = 93) and negative (n = 93) groups at random time points, disregarding the participants’ testing history. All of these samples were from participants not contributing to the training data, to avoid leakage from the training to the benchmark data. Using a NAAT‐based comparator as the reference standard, HRA had a positive percent agreement (PPA) of 96.6% (95% confidence interval (CI), 90.7–98.9%), an negative percent agreement (NPA) of 97.7% (95% CI, 92.2–99.4%) (Table 1). To assess cross‐reactivity, we used 33 additional influenza samples from subjects in the influenza vaccine trial cohort used for training. Two samples produced inconclusive HRA results, and the cross‐reactivity rate was 4/31 = 12.9% (95% CI, 4.2–30.7%) (Table 1). Overall, the cross‐sectional benchmark demonstrated a high concordance between HRA and NAAT results.

**TABLE 1:** Clinical evaluation, cross‐reactivity, and Host Response Assay early diagnosis

			Host Response Assay Interpretation		
Sample use	Sample type	Samples tested	Inconclusive	Positives	Negatives
Clinical evaluation	SARS‐CoV‐2 PCR positive	93	3	87	3
	SARS‐CoV‐2 PCR negative	93	4	2	87
Influenza Cross‐reactivity	H3N2 influenza	33	2	4	27
HRA early diagnosis	higher‐risk for NAAT early false negativity	15	0	10	5
	lower‐risk for NAAT early false negativity	8	0	0	8

We then performed a longitudinal benchmark by comparing HRA and NAAT repeated measures for the same participants over time (Figure 1E). The goal of this assessment was to explore whether HRA could anticipate SARS‐CoV‐2 diagnosis compared to NAAT. Due to the absence of a reference standard for SARS‐CoV‐2 diagnosis prior to NAAT positivity, we performed a validation study.^10^ We reasoned that some study participants were infected before their first positive NAAT result, but undetected due to low NAAT sensitivity early in infection. First, we defined groups of samples with higher and lower risk for NAAT early false negativity, based on phylogenetic and epidemiological evidence (Supporting information). Second, we compared HRA results in the two groups (Table 1; Figure 2). In the higher‐risk group, HRA was positive before NAAT in 10 of 15 participants (66.6%). In the lower‐risk group, HRA was positive in 0 of 8 participants (0%). The results support an earlier SARS‐CoV‐2 diagnosis using HRA as compared to NAAT (Fisher exact test, *p* = 0.0027).

**FIGURE 2:**
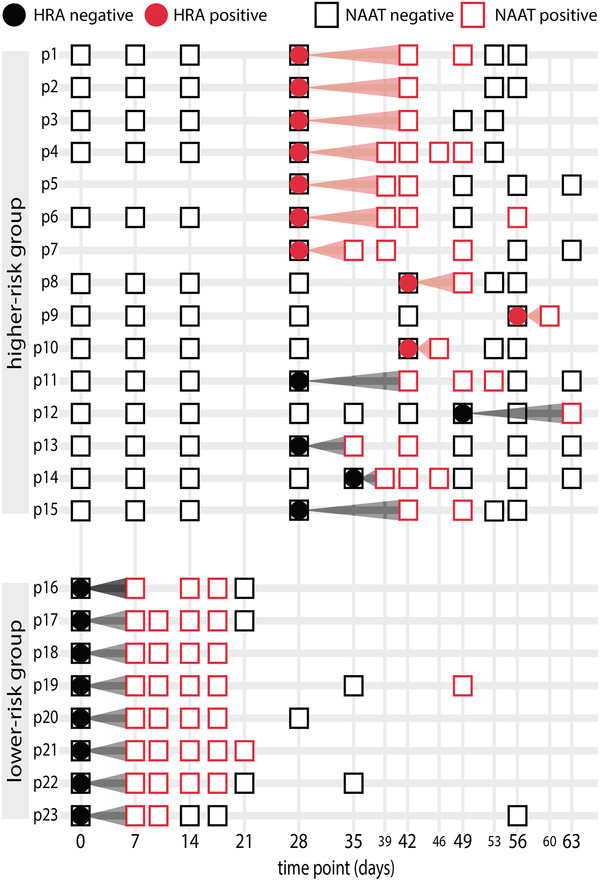
Detection of SARS‐CoV‐2 infection before positive NAAT result. To explore whether the HRA could accelerate SARS‐CoV‐2 diagnosis, we selected samples from two groups with higher and lower risk of NAAT early false negativity (Supporting information). Each row is a participant's history. Day 0 corresponds to arrival of the participant at supervised quarantine, which followed a 2‐week home quarantine, and day 14 corresponds to transfer to the basic training site, which had a high level of SARSCoV‐2 transmission. Blood samples were taken for each participant at the first time point available before the first positive NAAT result. At these time points, HRA and NAAT results were then compared. In the case of p12, a blood sample was not available before the first positive NAAT, and the previous available time point was assayed by HRA and evaluated for potential earlier detection. Symbols are times at which NAAT (rectangles) and HRA (solid circles) were performed. Colors are test results, either negative (black) or positive (red). In 10 of 15 participants, all in the higher‐risk group for NAAT early false negativity, HRA was positive at a time of a negative NAAT, demonstrating an accelerated diagnosis. These cases correspond to empty black rectangles enclosing solid red circles. The arrows connect the first NAAT positive result with the prior negative NAAT time point at which the HRA for early diagnosis was performed. They are red in case of HRA early diagnosis, or black in case of a concordant negative result by HRA and NAAT. In all participants in the lower risk group for NAAT early false negativity, HRA and NAAT gave concordant negative results

Limitations of our study include an unknown generalizability beyond young, healthy, male participants; some cross‐reactivity with influenza and possibly with other infections such as other coronaviruses; lack of knowledge of when SARS‐CoV‐2 exposure occurred, or of when NAAT would first turn positive with more frequent testing.

Since the beginning of the COVID‐19 pandemic, several diagnostic technologies have been proposed including surface‐enhanced Raman spectroscopy and field‐effect transistor‐based biosensors. Compared to these and other technologies, the main advantage of HRAs is the potentially higher sensitivity early in infection. This benefit should be assessed relative to the additional cost associated with blood draws. Although a cost‐benefit analysis was beyond the scope of our work, we envisage scenarios where using an HRA may be cost‐effective. These scenarios include, for example, hospitals and nursing homes where the need to ensure virus‐free environments is of critical importance.

In conclusion, our work provides the first implementation of a SARS‐CoV‐2 HRA and initial evidence that monitoring the host response can anticipate NAAT infection diagnosis.

## CONFLICT OF INTEREST

AC, JG, YG, VDN, NR, and SCS are coinventors of the Host Response Assay. JG and NR are employees of Fluidigm. AGL has nothing to declare.

## FUNDING INFORMATION

Defense Advanced Research Projects Agency (contract number N6600119C4022) has provided the funding for this work.

**Role of the Funder/Sponsor**: The funder had no role in any aspect of the study.

## DISCLAIMER

AGL is a military service member. This work was prepared as part of his official duties. Title 17, US Code §105 provides that copyright protection under this title is not available for any work of the US Government. Title 17, US code §101 defines a US Government work as a work prepared by a military service member or employee of the US Government as part of that person's official duties. The views expressed in the article are those of the authors and do not necessarily express the official policy and position of the US Navy, the Department of Defense, the US government or the institutions affiliated with the authors.

## Supporting information

Supporting Information
