# Supplementary material for: Earlier detection of SARS‐CoV‐2 infection by blood RNA signature microfluidics assay
Source: Clin Transl Discov. 2022 Jul 10;2(3):e47. doi: 10.1002/ctd2.47 (PMC9349572; doi:10.1002/ctd2.47)
Supplement: Supplementary file 1 — Supporting Information [file CTD2-2-0-s001.docx]

**Supplement**

**Sample collection**

There are two primary clinical sample types used in this work. The first set includes blood samples that had a paired nares swab PCR test result as part of the CHARM study. Institutional Review Board approval for CHARM was obtained from the Naval Medical Research Center (protocol number NMRC.2020.0006) in compliance with all applicable US federal regulations governing the protection of human subjects. All participants provided written informed consent. The second set of samples were influenza samples collected as part of the Influenza H3N2 Human Challenge Model (<https://clinicaltrials.gov/ct2/show/NCT03883113>) prior to the COVID-19 pandemic, and are assumed to be negative for COVID-19.

The CHARM and influenza samples are described in greater detail below.

*CHARM samples*

The SARS-CoV-2 positive and negative samples were part of a retrospectively collected cohort of samples from the longitudinal COVID-19 Health Action Response for Marines (CHARM) study. This included mostly male US Marine recruits, aged 18–20 years, who first quarantined at home for 2 weeks, unsupervised, prior to arriving at a Marine-supervised college campus or hotel for an additional two-week quarantine. At the end of the second quarantine, all recruits were required to test negative for SARS-CoV-2 by PCR testing before entering the basic training site.

Within 48 hours of arriving at the supervised quarantine location, recruits were given the opportunity to enroll in the CHARM study. Mid-turbinate nares swabs for SARS-CoV-2 PCR testing were obtained at multiple time points from the participants as well as blood samples collected in BD Biosciences PAXgene Blood RNA Tubes. These collection time points were not consistent for all participants and both nares and blood samples were not necessarily taken at the same time.

The nares swabs that were collected were tested for SARS-CoV-2 using quantitative PCR testing. Samples in viral transport media were maintained at 4 °C and all assays were performed within 48 hours of sample collection at high-complexity Clinical Laboratory Improvement Amendments (CLIA) certified laboratories using the US FDA authorized Thermo Fisher TaqPath COVID-19 Combo Kit (Thermo Fisher Scientific, Waltham MA). Lab 24 Inc (Boca Raton, FL) performed PCR testing from study initiation until the end of July 2020, and the Naval Medical Research Center (Silver Spring, MD) from August until the conclusion of the study in November 2020.

*Cross-reactivity influenza samples*

The cross-reactivity samples were obtained from an Influenza H3N2 human challenge model as part of a vaccination clinical trial (https://clinicaltrials.gov/ct2/show/NCT03883113). Each participant in the study was challenged with the influenza virus 28 days after receiving either placebo or the vaccine treatment. After the influenza virus challenge, each participant was assessed for the presence of the influenza virus by PCR at time points 2 days, 5 days, and 8 days after exposure. At the same time PAXgene blood samples were also collected.

**Definition of SARS-CoV-2 positive and negative cohorts used for algorithm training**

To train the host response assay classifier, we used a longitudinal comparator utilizing multiple nares swab test results taken at time points that were close together to determine the correct SARS-CoV-2 infection status of the participants for a particular time point. The comparator positive/negative status is defined by a strict set of rules that creates the Positive and Negative Cohorts defined below. To be part of the positive or negative cohort, the NAAT interpretation of the paired nares swab must have been available with the corresponding viral Ct values if the result interpretation was positive based on the NAAT.

For the training and validation of diagnosis (determination of false positive and false negative rates) only, the following definitions are utilized:

The Positive Cohort is defined as all blood samples that meet all the following criteria:

- The time point corresponding to the first positive nares swab is not the first available time point. This condition is motivated by the following considerations. To demonstrate that the host response assay turns positive before NAAT, we must restrict our analysis to participants that turn positive starting from at least the second available time point. This condition leaves us with the possibility that the host response assay may turn positive at an earlier time point compared to NAAT.
- The time point that the blood sample was taken was at or within 14 days of the first reported nares swab positive NAAT interpretation for the participant.
- The time point that the blood sample was taken does not fall after a time point with a discrepant interpretation. The time point that the blood sample was taken has:

(a) nares swab positive interpretation at the time point that the blood sample was taken

OR

(b) a nares swab positive NAAT interpretation both before and after the time point that the blood sample was taken within ±7 days. This is a positive-negative-positive sequence where the negative is determined to be a false negative and the clinical diagnosis is SARS-CoV-2 infected at the time point of the negative NAAT result.

The Negative Cohort is defined as all blood samples that meet all the following criteria:

- The blood sample has a negative nares swab NAAT interpretation at the corresponding time point the blood sample was taken. The blood sample is not part of the Positive Cohort (samples meeting criteria above are excluded from the Negative Cohort).
- The time point at which the blood sample was taken has neither a positive nares swab NAAT interpretation nor a discrepant nares swab NAAT interpretation within 14 days after the time point that the blood sample was taken when the time point is preceded by either no nares swab NAAT interpretation or another negative nares swab NAAT interpretation.
- The blood sample does not fall after the last nares swab NAAT positive interpretation and does not fall after any discrepant NAAT nares swab interpretation.

**Details on the selection of target transcripts**

The 41 targets were selected through a meta-analysis of transcriptional profiles of blood samples from SARS-CoV-2 positive and SARS-CoV-2 negative individuals. To minimize the risk of cross-reactivity with other viral and bacterial infections, the meta-analysis was based on an extensive, manually curated database of transcriptional profiles. Viral infections included: human coronavirus, influenza, BK virus, dengue virus, Epstein-Barr virus, Hepatitis E virus, human adenovirus, human herpesvirus 6, human parainfluenza virus, human rhinovirus, human T-Cell Lymphotropic Virus Type-1, respiratory syncytial virus. Bacterial infections included: *Burkholderia pseudomallei*, *Escherichia coli*, *Mycobacterium tuberculosis, Mycoplasma pneumoniae*, nontuberculous mycobacteria, *Staphylococcus aureus*, and *Streptococcus pneumoniae.*

In addition to viral and bacterial infections, the meta-analysis included non-infectious conditions acting as potential confounders, such as pneumonia, sepsis, obesity and diabetes.

The overall objective of the meta-analysis was to identify genes satisfying the following properties: 1) high discriminative power when comparing SARS-CoV-2 positive and SARS-CoV-2 negative individuals, and 2) marginal or no discriminative power when considering any other contrast in our database (e.g., influenza positive versus healthy controls). The identified panel consisted of the following 41 genes : ACTB; ARAP2; BANF1; B2M; CCNB2; CCNE2; CD4; CTNNAL1; DCTN1; EHD3; GUCD1; GUSB; HDAC1; IFI27; IFIT3; ITM2C; KLF1; LY6E; MPO; OAS2; OIP5; PADI4; PI4KA; PPM1F; PPP1R12B; PSMD12; ROCK2; RRM2; RTP4; SF3A1; SLC25A46; SLK; SMC4; SMPDL3A; TCEAL3; TFRC; TNFRSF17; TOP2A; TVP23B; TXK.

Five of these targets correspond to housekeeping genes (ACTB, B2M, GAPDH, GUSB, and TFRC) used for data normalization.

**Details on sample test using HRA**

Participant blood is collected by trained health care professionals in PAXgene tubes (BD Biosciences PN 762165). Total RNA is extracted from 400 μL of PAXgene preserved blood samples using RNAdvance Blood kit (Beckman Coulter PN A35604) according to the manufacturer’s instructions on Biomek i7 Automated Workstation. The quantity of the extracted RNA is estimated using Quanti-iT™ RiboGreen® RNA Assay Kit (Thermo Fisher Scientific PN R11490) using the Biomek FXᴾ Automated Workstation. The RNA is then normalized to 10 ng/µL by diluting with water. The RNA sample is further diluted to 2 ng/µL and used as an input for reverse-transcription and target pre-amplification. Pre-amplified material from each sample, together with qPCR reagents and the test assays, are then loaded into the 96.96 IFC (integrated fluidic circuit) (Fluidigm®) and processed on Juno™ (Fluidigm). The IFC is then transferred to Biomark™ HD (Fluidigm), where qPCR is performed, and the Ct values are recorded. The Ct values are then converted to a sample call by the interpretive software.

**Details on the interpretation algorithm**

The interpretation algorithm consisted of two primary parts: (A) normalization for run-to-run variability and (B) prediction modeling to make the interpretation.

Normalization of the Ct values was incorporated to create a more reproducible model. This was based on historically trained reference values established based on the process controls. The reference values must be first established up front. These reference values were then used on subsequent runs using the same process control lot to determine if there is a run assay bias difference observed between the historic reference values and the Ct values from the Process Controls on the run. This bias was then used as a correction that is applied to all samples on the run prior to interpretation.

After correction of the Ct values, the interpretation predictions were made based on a two-step process. The first step utilized a logistic regression classifier trained on CHARM COVID-19 positive and negative samples defined above (see section *Definition of SARS-CoV-2 positive and negative cohorts used for algorithm training*), and H3N2 influenza A samples treated as COVID-19 negative samples. From the full set of training samples, bootstrap subsets were used to train an additional 50 models. These additional models were used to check the consistency in calls. If there was low confidence in the interpretation because enough of the subset models yielded discordant interpretations, the final interpretation was changed to inconclusive. The second step used a second logistic regression classifier, but trained only using CHARM COVID-19 positive and influenza samples. Only the samples that gave a positive or inconclusive interpretation in the first step were processed through the second step, which changed calls from positive or inconclusive to negative based on a probability threshold.

The logistic regression algorithms were optimized through cross-validation using a training set of 245 SARS-CoV-2 positive, 296 SARS-CoV-2 negative, and 63 influenza samples. These training samples were randomly extracted from the corresponding cohorts. The final model was validated using 93 SARS-CoV-2 positive, 93 SARS-CoV-2 negative, and 33 influenza samples randomly selected from the respective cohorts. The validation samples were taken from participants not used for algorithm training.

**Definition of a higher-risk and a lower-risk group for NAAT early false negativity**

As a higher-risk group for NAAT early false negativity, we selected samples at the time of a negative NAAT from 15 participants following at least two weeks in basic training during a period of high SARS-CoV-2 transmission^2^ who had a first positive NAAT within a subsequent two weeks. As a lower-risk group for NAAT early false negativity, we selected 8 samples associated with a negative NAAT on arrival in quarantine and a subsequent positive NAAT. A total of 7 of the 8 of these participants had phylogenetic or epidemiological evidence consistent with SARS-CoV-2 infection occurring after their initial negative NAAT. Among the lower-risk arrival group samples, four of the participants had definite and two had probable virus sequence identification as belonging to a transmission cluster occurring after arrival at the quarantine site and four of the participants had SARS-CoV-2 infected roommates, which was identified as the highest epidemiological risk factor for being infected at the quarantine site[^2^](https://sciwheel.com/work/citation?ids=10920381&pre=&suf=&sa=0).
